# Adhesion Prevention in Gynecologic Surgery: Guidance and Clinical Experience

**DOI:** 10.3390/jcm13247517

**Published:** 2024-12-10

**Authors:** Ibrahim Alkatout, Rudy Leon De Wilde, Jörg Herrmann, Rüdiger Klapdor, Ivo Meinhold-Heerlein, József Mészáros, Alexander Mustea, Peter Oppelt, Julian Maria Pape, Sebastian Daniel Schäfer, Markus Wallwiener, Bernhard Krämer

**Affiliations:** 1 Department of Gynecology and Obstetrics, University Hospital Schleswig-Holstein, Campus Kiel, 24105 Kiel, Germany; julianmaria.pape@uksh.de; 2 Department of Gynecology, Carl-von-Ossietzky University, 26121 Oldenburg, Germany; rudy-leon.dewilde@pius-hospital.de; 3 Department of Gynecology and Obstetrics, Weimar Hospital, 99425 Weimar, Germany; j.herrmann@klinikum-weimar.de; 4 Department of Gynecology and Obstetrics, Albertinen Hospital Hamburg, 22457 Hamburg, Germany; ruediger.klapdor@immanuelalbertinen.de; 5 Department of Gynecology and Obstetrics, University Hospital Giessen, 35392 Gießen, Germany; ivo.meinhold-heerlein@gyn.med.uni-giessen.de; 6 Department of Gynecology, Obstetrics and Reproductive Medicine, University Hospital Magdeburg, 39108 Magdeburg, Germany; jozsef.meszaros@med.ovgu.de; 7 Department of Gynecology and Gynecological Oncology, University Hospital Bonn, 53127 Bonn, Germany; alexander.mustea@ukbonn.de; 8 Department of Gynecology, Obstetrics and Gynecological Endocrinology, Johannes Kepler University Linz, Kepler University Hospital Linz, 4020 Linz, Austria; peter.oppelt@kepleruniklinikum.at; 9 Department of Gynecology and Obstetrics, Clemenshospital Münster, 48153 Münster, Germany; seb.schaefer@alexianer.de; 10 Department of Gynecology and Obstetrics, University Hospital Halle, 06120 Halle, Germany; markus.wallwiener@uk-halle.de; 11 Department of Women’s Health, University Hospital Tübingen, 72076 Tübingen, Germany; bernhard.kraemer@med.uni-tuebingen.de

**Keywords:** gynecology, adhesion prophylaxis, barrier gel, 4DryField^®^ PH, infertility, pelvic pain, laparoscopic surgery, minimally invasive surgery, open surgery, robotic surgery

## Abstract

Postoperative adhesions represent a major medical challenge and are associated with serious health and economic consequences. 4DryField^®^ PH (PlantTec Medical GmbH, Lueneburg, Germany) is a starch-based medical device designed both to prevent adhesions and for hemostasis. This paper explores methods to successfully apply it in gynecological surgery, leveraging the authors’ extensive clinical experience. We provide detailed insights into best practices that benefit most patients with conditions such as endometriosis, along with practical tips and guidance on optimizing application and dosage. Our real-world clinical experience across various indications, supported by published data, demonstrates significant patient benefits: reduced adhesion formation, better recovery, less pain, and improved fertility. Patient acceptance and satisfaction are notably high. The device can be applied to surgical wounds as a powder for hemostasis and transformed into a gel in situ or as a premixed gel when adhesion prevention is prioritized. Specific advantages for each method are demonstrated by case studies. When used correctly, 4DryField PH is safe and effective, especially for larger wound areas with a high risk of reoperation and adhesion formation and when pregnancy is desired. It offers great versatility due to its use as either in situ gel or premixed gel with different viscosities. Despite some remaining gaps in clinical evidence and ongoing studies, our personal clinical experience suggests significant benefits with minimal risks. Therefore, we have no concerns regarding the broad use of 4DryField PH in gynecology and other surgical disciplines. Future research should focus on patient-reported outcomes and health economic benefits to support reimbursement efforts.

## 1. Introduction

Intra-abdominal adhesions are abnormal fibrous attachments between tissues and organs. They are mostly caused by irregular peritoneal healing after surgical trauma; other contributing factors include infection or radiation [1]. Adhesions begin to form early after surgical trauma; fibroblast growth follows on the third and angiogenesis on the fifth day [2]. However, the underlying mechanism is not completely understood yet, and, although minimally invasive surgical techniques have been shown to reduce the risk of adhesion formation, they do not eliminate it [3,4,5]. In general, adhesion formation comprises a very common post-operative complication, occurring in up to 95% of patients regardless of the surgical site and procedure [6]. Among gynecological procedures, endometriosis surgery, ovarian cystectomy, myomectomy, and oncological surgery have been considered especially adhesiogenic, even when using minimally invasive techniques [7]. Abdominal hysterectomy was found to be a major cause of adhesion-related small bowel obstructions (SBOs), while laparoscopic or vaginal hysterectomy was not [8,9]. On the other hand, even with a minimally invasive approach, more than 80% of patients were found to develop adhesions after the resection of deep infiltrating endometriosis (DIE) [10]. Although postsurgical adhesions may remain asymptomatic, a clinically significant subset of patients will develop a wide range of complications, such as bowel obstruction or chronic pelvic pain, and they also increase the difficulty and operating times in the case of reinterventions [11,12,13]. Patients who suffer from adhesion-related disorders often require multiple hospital visits and surgeries. Accordingly, complications caused by adhesions and their subsequent treatment are costly and represent a significant health economic burden [4,5,7]. Furthermore, postsurgical adhesions are a leading cause of secondary female infertility: indeed, 20–30% of infertile women were found to have adhesions [1,3,14,15]. While women suffering from infertility often benefit from adhesiolysis surgery, the re-intervention itself poses a renewed risk for adhesions to reform.

Apart from peritoneal adhesions, intrauterine adhesions (IUAs)—commonly referred to as Asherman’s syndrome—represent a significant burden and belong to the main reproductive system diseases worldwide [16]. Symptoms include menstrual disturbances, cyclic pain, and reproductive disorders [16,17]. The main cause of IUAs is pregnancy-related curettage, but hysteroscopic myoma resection was also associated with IUAs in up to 78% of patients, depending on the number and location of myomas removed [18].

The most obvious and effective strategy for preventing adhesions is to avoid surgery. If this is not possible, gentle tissue handling and a short duration of surgery will help to reduce adhesion formation, and minimally invasive surgery should be favored whenever possible [1,19,20]. As the presence of blood promotes adhesion formation, meticulous hemostasis is essential [2,21]. Postsurgical rinsing with saline or Ringer’s lactate represents good clinical practice but is not sufficient to prevent adhesions from forming [22]. Therefore, this risk remains, and further strategies for preventing adhesions are required. Pharmaceutical approaches have also been introduced, including steroids for their anti-inflammatory effects, heparin for anti-coagulatory effects, the tissue-plasminogen activator for fibrinolysis, and promethazine for the anti-inflammatory effects. However, low efficacy and side effects are an issue [15,23,24]. Additionally, various physical adhesion barriers are commercially available and can be categorized into solids, gels, or liquids. The regeneration of the peritoneum and its mesothelial layer is completed within 5–6 days postoperatively [25,26]; hence, such barriers are required to remain in place for this critical period [15]. The most recent systematic review on the prevention of peritoneal adhesions after gynecological surgery compared the outcome of all adhesion barriers on the market based on randomized controlled trials (RCTs) with second-look surgery to directly evaluate adhesions in the pelvic/abdominal cavity. As already stated in previous reviews, inconsistent results were found for most barriers. Nevertheless, expanded polytetrafluoroethylene (ePTFE), hyaluronic acid, and modified starch (4DryField PH) showed promising results, with ePTFE and 4DryField PH achieving the greatest improvements [27].

The aim of the present paper is to add more insights into the neglected topic of adhesion prevention in gynecologic surgery. The focus is on sharing personal long-term experience with the adhesion barrier 4DryField PH, giving practical advice for applications in a broad range of indications and pointing out strengths and weaknesses.

## 2. Materials and Methods

**Figure 1:**
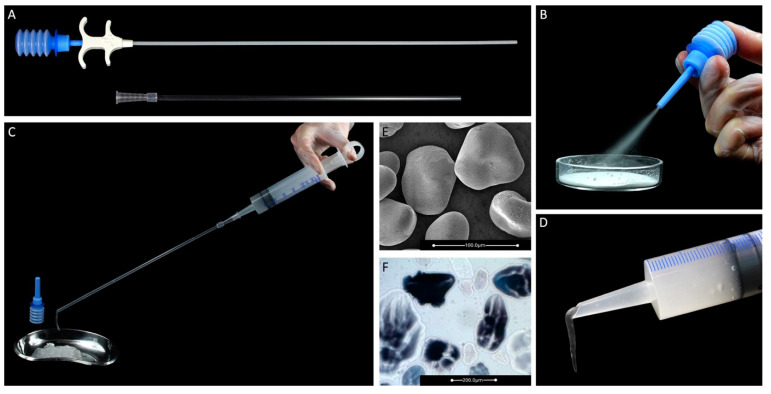
4DryField PH and its application: (**A**) the 4DFLap applicator with a flexible inner hose and rigid outer tube, (**B**) application of the powder directly from the bellows bottle, (**C**) application of premixed gel through the outer tube of 4DFLap, (**D**) application of premixed gel through a syringe, (**E**) powder microparticles before swelling (SEM image), and (**F**) powder microparticles after swelling (light microscopic image).

The starch-based 4DryField PH (Figure 1) serves two purposes: adhesion prevention and hemostasis. Both indications have been examined in many clinical studies, in the field of gynecological surgery, and beyond. It consists of sterile microparticles delivered in powder form; for hemostasis, it is directly applied to a wound surface—either from a bellows bottle or by using the 4DFLap applicator (in conventional and robotic minimally invasive surgery). Hemostasis is achieved by absorbing the liquid components of blood, thereby concentrating coagulation factors, as well as thrombocytes and the von Willebrand factor. The result is highly accelerated primary and secondary hemostasis [28], with clot firmness resembling native firmness even in 50% HAES-diluted blood [29]. After successful hemostasis, the white powder is gently irrigated with generous volumes of a sterile solution, such as saline or Ringer’s solution, until a gel has formed in situ. The gel then prevents intraperitoneal adhesions from forming by acting as a temporary physical barrier between surgical sites prone to form adhesions, hindering the formation of fibrin bridges. 4DryField PH is resorbed within about 7 days [27,30]. It is non-cytotoxic, and up to 1 g [30] or even 2 g in pediatric cardiac surgery [31] per kg of body weight can be used. It is known that a temporary, short-term increase in C-reactive protein (CRP) levels can develop in treated patients, which does not indicate the presence of infection but rather is the result of macrophage digestion of the powder, not associated with increased leukocyte concentration or body temperature [32]. If the main concern is to prevent adhesions, the powder can be applied directly as an extracorporeally premixed gel. At this stage, the authors wish to clarify that both powder and gel applications in varying ratios are feasible, depending on local tissue conditions and the surgeon’s discretion, as there is currently no evidence favoring one specific method over the other. Mixing ratios between 6 and 14 mL of isotonic saline solution per 1 g of 4DryField PH have been clinically used [33,34,35,36].

## 3. Results

We regularly use 4DryField PH in a multitude of indications with confidence. Our motivation for writing this article is to share our personal long-term experience using this product for adhesion prevention, to give advice for applying it in different indications, and to point out relevant dos and don’ts. In addition to its use for adhesion prevention, another strong benefit of the device is that its hemostatic abilities offer a less traumatic and tissue-conserving alternative to cauterization. Here, we will present clinical experience in highly relevant indications, which significantly benefit from the use of the product.

### 3.1. Adhesion Prevention After Endometriosis Surgery

The use of 4DryField PH is beneficial for patients who require surgery for endometriosis. Indeed, endometriosis affects 10–15% of women of reproductive age and may cause a variety of symptoms, including infertility and pelvic pain. Surgical treatment is often required and involves the excision or ablation of endometrial tissue. This bears certain risks, and adhesions develop in more than 90% of cases. The first RCT with 4DryField PH [23] analyzed its effectiveness as an adhesion barrier after endometriosis surgery. Only women with extensive endometriosis and DIE, requiring a definite final procedure via second-look laparoscopy, were included. The second intervention allowed for the direct analysis of extent and severity as well as the incidence of adhesions compared to a control group treated with saline flushing. In this study, the severity and extent of adhesions were significantly reduced by 85% in the intervention group compared to the control group. In addition, the incidence of adhesion formation based on the number of affected sites was significantly reduced by 53%. The follow-up [37] revealed that the pregnancy rate in the intervention group was also significantly higher. Furthermore, pain scores were lower, with the most striking improvements in cycle-independent pelvic pain and dysmenorrhea. As lower adhesion scores had already been shown, these outcomes could be linked directly to effective adhesion prevention. These remarkable results, when confirmed with larger cohorts, could provide the basis for clear recommendations in women with endometriosis and desiring pregnancy.

**Figure 2:**
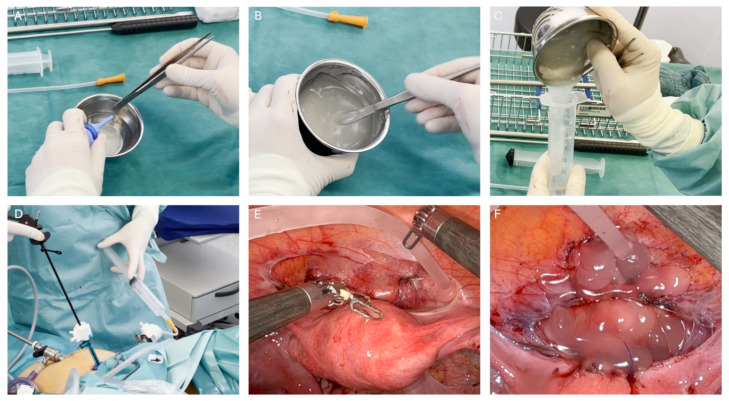
4DryField PH applied as a premixed gel in laparoscopic endometriosis surgery: (**A**) 3 g of 4DryField PH powder is sprayed from the bellows bottle into a 30 mL sterile solution. (**B**) Thorough mixing until a homogeneous gel is achieved. (**C**) Drawing up or filling the gel into a syringe. (**D**) Surgical setup with tubing inserted into the pneumoperitoneum. (**E**) After the removal of endometrial tissue, the gel is directly applied on the desired sites by grasping and guiding the tubing with endoscopic scissors. (**F**) All surgical areas are completely covered, and the gel reliably stays in place but does not stick too firmly.

Powder 4DryField PH has the benefit of first providing hemostasis and, after moistening, preventing adhesions, but the application of premixed gels with different viscosities is appreciated by the authors. Using the gel, surgical wound sites can be covered with reliable thickness, and it has been reported that the premixed gel is a convenient variant for larger peritoneal wounds [33]. For endometriosis surgery, we agree that the use of a premixed gel is favorable because it can be distributed with ease and controllably to all ablated sites and remains well in place during and after application. A clinical case with a step-by-step approach in using 4DryField PH as a premixed gel in endometriosis surgery is shown in Figure 2, in which 3 g of 4DryField PH powder was mixed extracorporeally with 30 mL of saline solution.

When preparing the premixed gel, the powder must be mixed until a homogeneous gel has formed. If kept in the sterile area and in a syringe to avoid evaporation, the gel stays stable for hours. Generally, a mixing ratio of 10 mL of sterile solution per 1 g of 4DryField PH is suitable for most requirements. If necessary, thicker or thinner gels can be used as well [33,34,35,36]. Thin gels with a mixing ratio of 1:10 or higher can be drawn up into a syringe, while thicker gels with a mixing ratio of 1:6 to 1:8 need to be transferred into a syringe with a spatula. As the powder forms gels even beyond a mixing ratio of 14 mL per 1 g of 4DryField PH, the premixed gels likely still absorb some liquid at the sites of application. Naturally, the cleanliness of all materials is key, and it should always be ensured that the gel being formed is free of blood as blood impedes the effectiveness of adhesion prevention. The 4DFLap outer tube can be used to apply the premixed gel. Extracorporeally mixed gel is often preferred for laparoscopic surgeries, especially for larger wound areas with no or with minor bleeding only. For larger areas, thinner gels might be useful (e.g., Blumhardt et al., 2018 [38]).

### 3.2. Preventing Adhesion Re-Formation After Adhesiolysis Surgery and Neuropelveology

**Figure 3:**
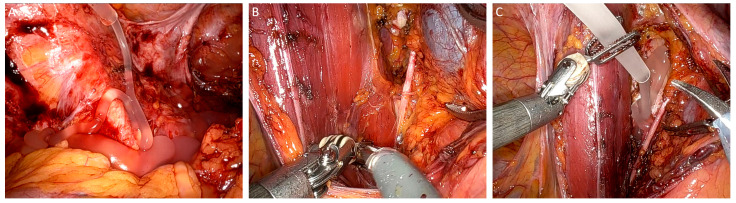
4DryField PH applied as a premixed gel in laparoscopic adhesiolysis surgery (**A**) and neuropelveology (**B**,**C**).

Patients undergoing adhesiolysis surgery often have a long history of chronic pain or infertility. Some patients require the procedure multiple times due to recurring adhesions. In such difficult clinical cases, repeated adhesiolysis alone does not solve the problem as detached adhesions are predilection sites for adhesion reformation. In a study on patients undergoing adhesiolysis and a subsequent second look laparoscopy, it was shown in the control group that adhesiolysis alone did not decrease the extent of adhesions and even increased the adhesion severity [39]. In contrast, the intervention group treated with 4DryField PH showed a significant 75% reduction in both adhesion severity and extent—although the initial mean adhesion severity and extent had been higher in this group. While patients were not completely free of adhesions after applying 4DryField PH, it still provided significant relief in this challenging clinical situation. For adhesiolysis, premixed gels are used, as hemostasis is typically not a concern. A mixing ratio of 1:10 is used, with 3 to 5 g, depending on the extent of present adhesions [39]. A clinical case is shown in Figure 3A. Another indication with high adhesiogenic potential is neuropelveology [40,41]. Although we have started to use 4DryField PH only recently, the first results look promising, and it appears to be another innovative area of application for the future (Figure 3B,C).

### 3.3. Ovarian Cysts: Hemostasis and Adhesion Prevention

Bipolar electrocoagulation or cauterization is a standard procedure for hemostasis in ovarian surgery but inevitably causes tissue damage, which in turn results in a reduction in the ovarian reserve, as indicated by a diminished concentration of the Anti-Müllerian hormone (AMH) [42,43]. 4DryField PH has been shown to represent an immediate and less traumatic alternative to cauterization for the purpose of hemostasis [44,45,46] and in patients with unilateral ovarian tumors or cysts undergoing laparoscopic tumor/cyst enucleation. There was no postoperative reduction in the ovarian reserve, whereas it was significantly reduced in the control group [46]. Deep ovarian endometriosis or endometrioma represents a special type of ovarian cysts. The surgical removal of larger and growing lesions is recommended to avoid damaging ovarian tissue. In their study on endometrioma infertility surgery, Torres-de la Roche et al. found that intraoperative periovarian coagulation was no longer required when using 4DryField PH, which improved the preservation of tubo-ovarian function [45]. In addition to conserving fertility, 4DryField PH also strongly reduces the likelihood of the ovary being adherent to surrounding organs and thus provides the further protection of fertility [45].

Therefore, ovarian surgery, like the removal of ovarian cysts, benefits from the use of the powder to arrest bleeding in a gentle and tissue-conserving way. Coagulation can often be avoided or at least reduced, thereby helping to maintain fertility. Three grams of powder is typically sufficient for ovarian surgery and to cover the bed of the enucleated cyst.

The in situ gel obtained after the obligatory gelation of the powder by drizzling with a sterile solution subsequently provides adhesion prevention, serving two purposes with one application. When using the powder, it is pivotal to completely transform it into a gel because only the gel prevents adhesions and subsequently also facilitates biodegradation by macrophages. A generous volume of a suitable sterile solution should always be used to completely transform the powder into a gel. However, the powdered layer should not be soaked with peritoneal fluid because the fibrin contained in peritoneal fluid may contaminate the gel, potentially providing an insufficient barrier for adhesion prevention. A clean, sterile solution, on the other hand, leads to a fibrin-free gel and effective adhesion prevention. Here, all required areas should be evenly covered with powder, without any thick accumulations. Thick accumulations of powder should be avoided as they will require more time and thoroughness to be transformed into a gel, and thick accumulations of dry powder might also exacerbate resorption by the body. Drizzling of the powder with the sterile solution should always be gentle as flushing with high volumes or high pressure might rinse the powder and gel off.

The homogeneous application of powder requires skill and a certain learning curve. If the powder appears too thick in certain areas, clean air, e.g., from the emptied bellows bottle, can be blown through the applicator to better distribute and thin the layer of powder. However, this needs to be performed carefully to avoid widespread dispersal of the powder—which would require an increased effort in drizzling with a sterile solution. On the other hand, enough powder needs to be used—especially in cases of stronger bleeding—because powder soaked with blood or wound exudate will not prevent adhesions. Based on experience, a white appearance of the upper powder surface indicates that bleeding was arrested. And, when turned into a gel, a sufficient barrier for adhesion prevention is provided. If there is blood in the powder, additional powder needs to be applied.

**Figure 4:**
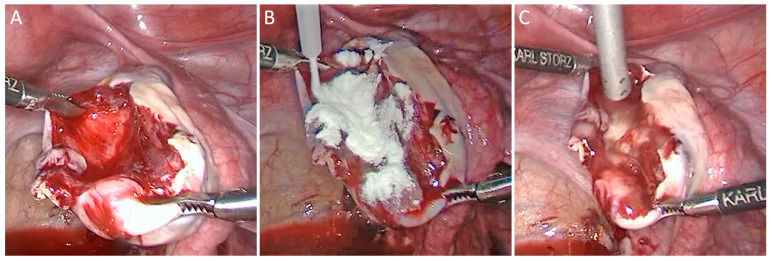
4DryField PH applied as a powder for hemostasis with subsequent gel transformation for adhesion prevention in ovarian cyst surgery: (**A**) Ovary after resection of the cyst. (**B**) Applying the powder for tissue-conserving hemostasis. (**C**) Transformation of the powder into a barrier gel for adhesion prevention by drizzling with sterile saline.

A clinical case with a step-by-step approach in using 4DryField PH as a powder in the surgery of ovarian cysts is provided in Figure 4. After hemostasis is achieved, the gel prevents the ovaries from adhering to surrounding structures.

### 3.4. Ovarian Carcinoma: Hemostasis and Adhesion Prevention

**Figure 5:**
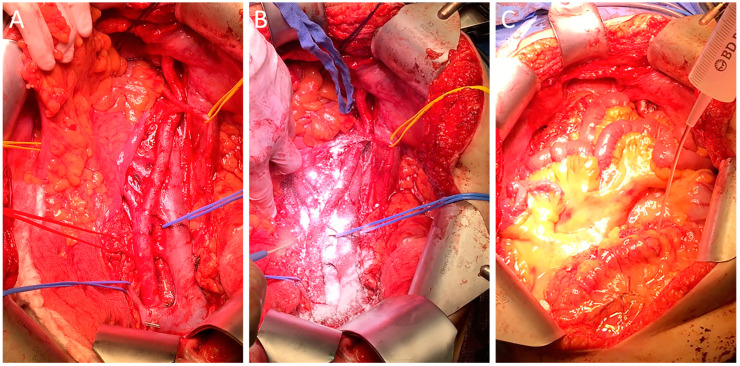
Application of 4DryField PH in open ovarian carcinoma surgery: (**A**) Site before product application. (**B**) Powder application directly from the bellows bottle for lymphostasis. (**C**) Application of a premixed, thin barrier gel for adhesion prevention.

4DryField PH is also applied in various oncologic gynecological procedures, which often bear a higher risk of adhesion formation due to their complexity. Malignant disease and previous radiotherapy were shown to be independent risk factors for adhesion-related readmission and abdominal reoperation after gynecological surgery [47]. While data from clinical studies with 4DryField PH do not exist yet, our experience shows that it offers gentle but effective hemostasis and adhesion prevention. The surgery of ovarian carcinoma also benefits from the use of the powder, combined with a thin gel for adhesion prevention (Figure 5).

### 3.5. Cesarean Section

**Figure 6:**
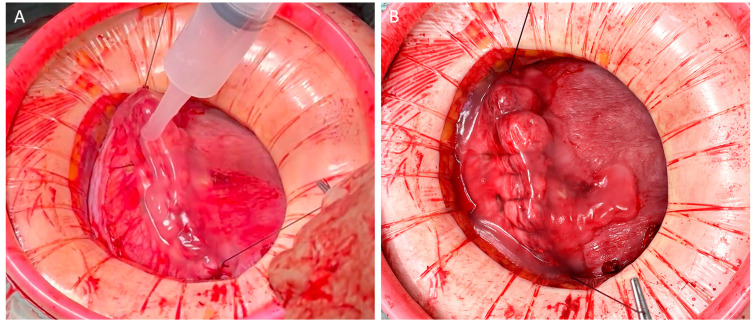
4DryField PH applied as a premixed gel for adhesion prevention in cesarean section: (**A**) Application of the premixed 4DryField PH gel (1:10 gel with 3 g of powder mixed with 30 mL of a saline sterile solution). (**B**) Site after gel application.

Cesarean delivery rates have increased rapidly in recent years and are now the most common surgery in women of reproductive age. In one study, adhesions were found in 37.5% of women with a history of cesarean sections [48]. Repeated cesarean delivery represents increased risks because of adhesions such as placenta previa, placenta accrete, surgical injury to adjacent pelvic organs, and excessive bleeding [49,50]. Increased incision-to-delivery times were found for women with adhesions due to previous cesarean sections, and the adhesion score was found to correlate with time [50]. To prevent the formation of adhesions after cesarean surgery, the use of 4DryField PH has proven useful in our clinical practice, and, today, the use of a premixed gel is preferred. For cesarean sections, the premixed gel is mostly preferred; we commonly use 3 g mixed with 30 mL of a sterile solution (Figure 6).

### 3.6. Hysterectomy

**Figure 7:**
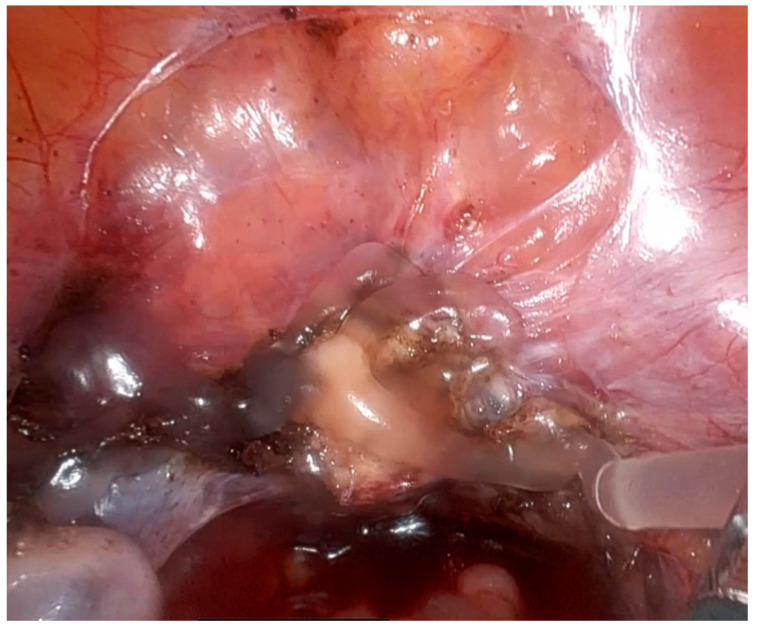
4DryField PH applied as a premixed gel for adhesion prevention in hysterectomy.

Hysterectomy is the second most common gynecological procedure, performed more than half a million times yearly in the United States alone with about 30% of women undergoing the procedure by the age of 60 years. Approximately 70% of procedures are performed for benign indications such as leiomyomas, adenomyosis, fibroids, and endometriosis [51,52], and total hysterectomies involve the removal of the uterus and cervix. Radical hysterectomy is performed in women with cancer and represents the more extensive procedure, also including the top part of the vagina, ovaries, fallopian tubes, lymph nodes, lymph vessels, and surrounding tissue [53]. Complications that are potentially related to adhesion formation include SBOs, ileus, or chronic pain [9,54,55]. Malignancy was shown to be an independent risk factor for SBO [9]. The risk of readmission for adhesion-related complications was shown to be 10-fold higher after hysterectomy than after uterus-sparing surgeries, with a high rate of reoperations being required. Rates for laparotomy and laparoscopy were found to be similar, but only vaginal hysterectomy showed lower rates [47]. To improve clinical outcomes after hysterectomy, we recommend adhesion preventive measures. For this purpose, we use 4DryField PH as a premixed gel, as shown in Figure 7. Depending on the extent of the procedure, often determined by whether the surgery was due to benign or malign reasons, 3 or 5 g are used, premixed with 30 or 50 mL of a sterile solution to achieve a mixing ratio of 1:10.

### 3.7. Intrauterine Procedures: Operative Hysteroscopy

Hysteroscopy is performed for curettage, septal incision, the removal of intrauterine myoma, or the removal of adhesions in cases of Asherman’s syndrome and presents a significant risk for the formation or renewed formation of IUAs. The rates of renewed adhesions are high, and, after adhesiolysis, adhesions recurred in 76% of patients [56]. The development of IUAs caused by operative hysteroscopy can cause infertility and increase the risk of recurring miscarriages [57]. Furthermore, IUAs can lead to the partial or total obliteration of the uterine cavity, often causing dysmenorrhea, hematometra, and severe pelvic pain [58]. Therefore, applying anti-adhesive measures is of great importance in operative hysteroscopy. In a recently published study, patients undergoing adhesiolysis for Asherman’s syndrome received either 4DryField PH or Hyalobarrier gel, a hyaluronic acid-based agent with clinically proven effectiveness in preventing IUAs [36]. The 4DryField PH gel was premixed from 18 mL of saline solution and 3 g of 4DryField PH. Both devices effectively prevented the recurrence of IUAs, but adhesion reduction with 4DryField PH was higher, particularly in patients with severe IUAs, and the pregnancy rate during follow-up was 50% in the 4DryField PH group vs. 20% in the Hyalobarrier group [36]. Accordingly, premixed 4DryField PH gel is a very promising adhesion barrier for preventing IUAs.

**Figure 8:**
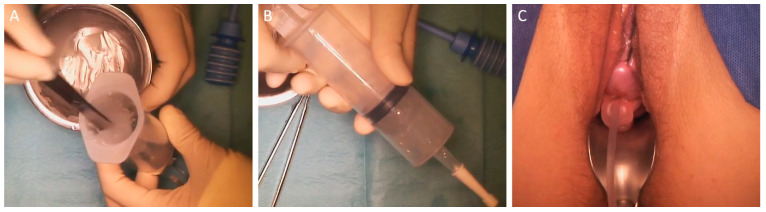
4DryField PH applied as a viscous, premixed gel for adhesion prevention in hysteroscopic surgery: (**A**) The thick gel (here, 3 g of 4DryField PH with 24 mL saline solution) is filled into a syringe with a spatula. (**B**) The syringe is connected to a suction catheter. (**C**) The gel is injected until excess extrudes from the cervix. The required amount of gel varies depending on the size of the uterus.

For intra-uterine applications, a premixed 4DryField PH gel is generally used. A mixing ratio of 1:6 to 1:8, typically an 18–24 mL sterile solution mixed with 3 g of powder, has proven beneficial for this indication. At this thick consistency, drawing up the gel into a syringe is no longer possible, and a spatula needs to be used to transfer the gel (Figure 8A). For application, a suction catheter is well suited because of its flexibility (Figure 8B). Any excess gel will simply be discharged, and the amount used will depend on the size of the uterus (Figure 8C).

## 4. Discussion

In our experience, patient acceptance and satisfaction after the application of 4DryField PH is very high. Patients experience less pain after surgery, and they recover well. Other clinical observations include a low volume of drainage and a generally enhanced healing process. It also appears that tissue elasticity is maintained well, and any scars that form appear to be “softer”. Positive effects on healing have been observed, but the exact mechanism has not been understood yet. In trials with second-look laparoscopy, a correlation between reduced pain and reduced adhesions was observed. This implies that, indeed, the adhesion preventive capabilities of 4DryField PH led to less pain. While there may still be gaps in clinical evidence and results are still pending from ongoing clinical studies, we believe, based on our clinical experience, that the patient benefit from preventing adhesions is so significant and the risks so low that a more widespread use should already be implemented. While using 4DryField PH routinely in all cases has many advantages, there are specific reasons for using this product when wound areas are large, and a generally expected high risk for adhesion formation or renewed formation is present. In addition, the localization and proximity of different wound surfaces to one another should be considered.

The product is highly versatile as it can be applied as a powder with subsequent gel transformation through drizzling with a sterile solution or as premixed gels with consistencies adapted to individual needs and preferences. Other products are typically delivered in just one consistency. Furthermore, refrigeration or lengthy preparation is not required. While it can be applied selectively where bleeding occurs and where adhesions are most likely to form, it is equally possible to treat larger areas. For the on-point, reliable treatment of specific smaller regions, a thicker consistency is considered useful. A particular benefit, however, is that a thin gel can easily be applied to large areas, when extended areas are at risk for adhesion formation. Gels can be prepared within a wide range of mixing ratios between approximately 6 and 14 mL of sterile solution per 1 g of powder.

The required amounts of material depend on the type of indication and the area of surgical sites requiring treatment. According to the manufacturer, 5 g of product will cover an area of at least 125 cm^2^. For example, in the RCT on endometriosis surgery using an in situ gel, the mean amounts used were 3.2 g of powder (range of 1–5 g) [23]. When using a premixed gel, a mixing ratio of a 10 mL sterile solution per 1 g of 4DryField PH powder works well for most approaches. Indeed, 3 or 5 g of the product will provide a sufficient volume for most cases. For hysteroscopy, a thicker gel with a lower mixing ratio of about 6–8 mL of saline solution per 1 g of 4DryField PH is used. Finally, each surgeon can choose the preferred consistency and adapt it to the individual clinical situation.

The ideal thickness of the gel layer required to effectively prevent adhesions is a commonly discussed question, regardless of whether an in situ or a premixed gel is used. It appears that the gel film directly adhering to the wound surface represents a crucial barrier for effective adhesion prevention. Using larger amounts of gel does not present a problem; any excess gel can simply be discarded or left in situ for biodegradation. Some surgeons evenly line the whole bowel area with a thin layer of premixed gel to prevent adhesions everywhere, saving the effort to apply the gel specifically where it is deemed to be required and making sure no place is missed. Blumhardt et al. have described this approach previously and used low viscosity gels with mixing ratios between 60 and 70 mL of saline per 5 g of powder to treat large areas at risk for adhesion formation in the abdomen [38]. The thin gel facilitated the homogeneous and convenient distribution on the intestinal loops in cases of extensive dissection of intestinal adhesions with chronic obstruction or acute intestinal obstruction with ileus. No negative influence on suture healing was found. In general, thin gel that migrates to areas where it was not applied, e.g., upon movement of the patient, is not considered critical and would also be degraded within about 7 days.

In addition to providing hemostasis and preventing adhesions, it appears that 4DryField PH also has a lymphostatic effect and might be able to prevent lymphocele formation. Karsch et al. showed that, after lymphadenectomy following retropubic prostatectomy, the use of 4DryField PH effectively provided hemostasis and stopped wound oozing [59]. Furthermore, the incidence of late lymphoceles and lymphoceles requiring treatment was reduced by half, and drain loss was reduced in these patients compared to controls. A combined hemostatic and lymphostatic effect is likely due to the similarities of lymph with plasma and the clotting factors that lead to similar albeit slower coagulation. Gynecological oncologic surgeries, such as the removal of ovarian tumors, often require lymphadenectomy in the lesser pelvis. Therefore, an additional lymphostatic effect of 4DryField PH might positively influence the healing process. However, this potential effect needs to be confirmed in larger studies in gynecological surgery.

The indications of other starch-based hemostats have recently extended to include adhesion prevention. However, in a direct comparison of 4DryField PH with HaemoCer PLUS and StarSil, only 4DryField PH was able to effectively prevent adhesion formation in an established rat model, demonstrating that starch-based hemostats do not generally also provide effective adhesion prevention [60]. Previously, it was shown that another starch-based device, Arista AH, did not statistically significantly reduce adhesion formation, whereas 4DryField PH did [61]. Therefore, differences between the starch-based devices appear to exist. To the best of our knowledge, 4DryField PH is the only starch-based adhesion barrier that contains citrate in its final form. During natural coagulation, fibrinogen is cleaved to fibrin. Fibrin then polymerizes and forms polyfibrin, which provides the basis for wound closure. The formation of adhesions is based on polyfibrin strands as well. Citrate is known to inhibit the cleavage of fibrinogen to fibrin and therefore influences both hemostasis and adhesion formation. A possible explanation underlying the highly effective hemostasis despite the presence of citrate is that dry powder is used for hemostasis. The influx velocity of liquid is very high in the timeframe required for hemostasis; citrate ions cannot diffuse out of the powder so that polyfibrin can form to build the coagulate. When the powder is turned into a gel, the influx of liquid is very low, and citrate ions can freely diffuse—and thus further support adhesion prevention by hindering fibrin bridges from forming. Potentially, this could be one explanation for the superior adhesion prevention capabilities that have been described. Another factor that has been discussed in several publications is the longer degradation time of 4DryField PH (7 days) compared to other starch-based adhesion barriers (1–3 days), the latter ones apparently being resorbed before peritoneal healing is completed [5,27,60].

Another potentially overlooked aspect is that, due to its nonphysiological sodium and chloride concentrations and low pH, normal saline may not be the ideal fluid for 4DryField PH gelation. Saline has been reported to cause hyperchloremic acidosis [62] when used for infusions. When used for peritoneal lavage, it promotes the exfoliation of mesothelial cells in in vitro models [63] and might represent a risk factor for adhesion formation on its own [64,65]. The administration of normal saline might also stimulate the implantation of ovarian cancer cells onto the omentum [62]. Buffered solutions with physiological concentrations of chloride, such as Ringer´s solution, can be used with 4DryField PH as well and might therefore be better suited and enhance adhesion prevention when used in combination with 4DryField PH. However, the effectiveness of 4DryField PH has mostly been shown in combination with saline solution, and the overall volumes used are generally low. Nevertheless, further examination of this topic would be of interest; likewise, it would also be of interest to investigate whether the use of humidified CO_2_ can lead to enhanced adhesion prevention in combination with 4DryField PH [2].

### 4.1. Tips and Tricks for Successful Application

Based on our combined clinical experience, Table 1 provides some general advice and tips on the use of 4DryField PH to facilitate and optimize application.

### 4.2. Limitations

So far, only one side effect has been reported for 4DryField PH: in some cases, a temporary, short-term increase in CRP levels has been observed [32,66]. In both studies, the maximum post-operative CRP level was significantly higher than in the control group, whereas leukocyte concentration and body temperature did not differ between groups. No signs of infection were detected in any of the patients, and CRP levels spontaneously dropped to normal values within a few days. No side effects or complications were observed in both groups. In second-look surgeries performed for other diagnoses, no remnants of 4DryField PH or any peritoneal inflammatory reactions were observed. Ziegler et al. [32] concluded that the CRP level increase observed in some patients is not indicative of an infection but the result of macrophage digestion of powder particles and not associated with increased leukocyte concentration or body temperature.

In addition, some clinicians have expressed concerns regarding the use of the dry powder due to its biocompatibility and potential side effects. In isolated cases, it was found that in situ degradation can be substantially delayed. In the case of a 28-year-old woman requiring emergency hysterectomy due to serious post-partum hemorrhage and associated consumption coagulopathy, conservative measures such as electro-cautery, suturing, and packing with sterile towels had failed to provide sufficient control of the diffuse bleeding. Rhesus factor-compatible donor blood was no longer available; finally, a very large dose of 25 g of dry 4DryField PH powder was applied to the lesser pelvis to stop the diffuse bleeding. After the patient´s circulation had stabilized, a drain was inserted, and the abdomen was closed. In situ transformation of the powder to a gel was omitted in this emergency. The patient survived with an intraoperative blood loss of 4.2 L. After 18 months, having undergone two further surgeries for incisional hernia repair and appendectomy in the meantime, the patient presented with a peritoneal cyst in the lesser pelvis and also adhesions. Upon removal of the cyst, a whitish layer with a shiny surface without adhesion formation was identified in the lesser pelvis at the peritoneum, bladder peritoneum, and vaginal stump. Histology revealed the presence of foreign material by showing a positive periodic acid-Schiff (PAS) reaction, which was thought to possibly be a residue of the earlier treatment with 4DryField PH. Interestingly, some lymphocytes were present, but neither granulocytes nor fibrous capsulation was found. Considering the amount of the foreign body, the negligible inflammatory response, and lack of necrosis and granuloma formation are notable. The presence of macrophages in the histological outcome further indicates that degradation was ongoing and might have eventually completely cleared the starch. The area where residual starch was found showed an intact peritoneum, and, most importantly, no signs of malignancy or other clinical symptoms were observed. While the glove powder containing starch has been described to induce granuloma formation [67,68], it is known that potato starch is typically rapidly degraded by alpha-amylase and glycol-amylase enzymatic activity and macrophages [69,70]; thus, the described cases might be attributable to impurities. After several purification steps, 4DryField PH consists of highly purified starch. Potentially, a lower amylase enzyme activity owing to the genetic disposition of the patient might have been the reason for slow degradation. Additionally, the very large dose and lack of transformation of the powder into a gel may have contributed to residual material. Nevertheless, 4DryField PH was able to arrest the critical bleeding in this case, and the remaining material did not cause any clinical complications.

Ziegler et al. described the case of a 71-year-old woman with serometra and endometrial hyperplasia in whom the anterior wall of the uterus was perforated during the hysteroscopic resection of submucosal polyps and a fractional curettage [35]. During the immediately initiated laparoscopy, active bleeding from the 1 cm wound was stopped by applying dry 4DryField PH powder, so that coagulation or suturing could be successfully avoided. The powder was not transformed into a gel. Patient recovery after surgery was satisfactory. Nine weeks later, a laparoscopic hysterectomy with bilateral salpingo-oophorectomy for endometrial carcinoma was performed. At this second surgery, the pelvic organs were found to be free of adhesions, and the area of previous perforation was covered with an apparently normal peritoneum. In the right Douglas pouch, a small, 0.5 mm, white granuloma with a foreign body reaction and regressive calcification was found and excised. Although a direct connection with 4DryField PH could not be established, the small granuloma might possibly have been caused by a large amount of dry powder that was not transformed into a gel.

It needs to be stressed that the powder should always be completely transformed into a gel, with no powder residues left. Dry powder will inevitably cause some desiccation—potentially leading to tissue lesions or even minor local tissue necrosis. Thus, after hemostasis is achieved (typically after 30–60 s), the powder should not be left in place any longer than necessary: drizzling with a sterile solution is required until full gelation and formation of the in situ gel. It has been noted that, when transforming thick layers of powder, some sorts of macroparticles remain that still contain powder. It is therefore conceivable that powder that was not completely transformed into a gel remains at the sites of application with delayed degradation; however, such cases are very rare. Most clinical study results so far report on the use of situ gel, highlighting that this method of application works well and is safe, further supported by our own clinical experience. Also, in several studies with second-look interventions after 1 week or longer, it was stated that no remnants of powder were detected [23,33], even with intervals to second look as short as 1 week [39]. However, dry powder left in situ might rarely become a problem if large amounts are used. Even when carefully applying the powder, an even distribution is not always possible, and certain areas unavoidably get a thicker coverage than others, which might lead to slight differences in the amount of powder applied [23]. Similarly, the volume of saline solution varies, and a standardization of in situ gel application is hardly possible [23]. Based on the available data, it is not clear if a thicker layer of the barrier—as it has been depicted, e.g., by Ahmad and Crescenti [66] and Ziegler and De Wilde [39]—is more effective than a thinner one. Overall, preferences vary individually, and, based on current knowledge and experience, the effectiveness of the in situ gel and extracorporeally mixed gel is considered clinically equivalent in preventing adhesions. Although no clinical studies directly comparing the efficacy of both ways of the application are available, the premixed gel has several advantages over the in situ gel, mostly the convenient handling, user-friendliness, and reproducibility. Application is easy, the gel is firm, and it adheres well to wound surfaces, while the drizzled powder has been observed to become runny. It can be applied slowly and controllably and stays where placed, even in challenging situations. The powder particles are distributed evenly in the gel, and the amounts of powder and sterile solution can be adapted to individual needs. Also, it is not necessary to take the additional step of carefully drizzling all over the powdered area, which saves time. It has further been observed that the CRP level increase described in some cases [32] develops less often when a premixed gel is used, probably due to the facilitated macrophage degradation of the premixed gel with its evenly distributed and sufficiently moistened powder particles.

Although the safety of 4DryField PH has been confirmed in many studies, every introduction of foreign material into the body needs to be considered carefully and weighed against the clinical benefit. In addition, the cost–benefit ratio of such a measure must be positive. Systematic results for the healing of fresh bowel anastomoses have only recently been published: Liu et al. found no differences for blood loss, operating time, and postoperative complications after colorectal surgery compared to a control group. Furthermore, adhesion scores were significantly lower in the group treated with 4DryField PH vs. controls [71]. In particular, no cases of anastomotic leakage occurred, contrasting the increased rates of anastomotic leakage described for other adhesion preventive agents such as Seprafilm^®^ [72,73,74] or complications such as SBO described for a 4% icodextrin solution [75]. The positive safety results were confirmed in a retrospective study, including 157 patients after rectal surgery and primary anastomoses, where 4DryField PH again did not increase the rate of anastomotic leakage [76].

## 5. Conclusions

The use of 4DryField PH has shown significant benefits in gynecological surgeries, and we consider it a valuable tool in specific situations. Our multiple years of clinical experience, supported by published clinical data, highlight its effectiveness in reducing adhesion formation and supporting better patient recovery, less pain, and improved fertility. Due to the compelling results and high safety, we have confidence regarding the broad implementation in clinical practice, among the various clinical disciplines. Because of its advantages, the premixed gel should be used when hemostasis is not required.

Looking ahead, the evidence from initial smaller scale studies should be solidified by well-designed trials with larger patient cohorts. Several such studies are currently ongoing, such as the ASPIRE study, a multicenter RCT in endometriosis surgery that focuses on patient-reported outcome measures, such as quality of life and fertility (https://drks.de/search/en/trial/DRKS00033730 (accessed on 1 October 2024)). Another multicenter RCT in women undergoing their first cesarean sections evaluates, for example, adhesion formation and pain scores (https://trialsearch.who.int/Trial2.aspx?TrialID=NL-OMON23062 (accessed on 1 October 2024)). Additionally, the first multicenter RCT on the application of 4DryField PH in different hysteroscopic procedures is currently underway, evaluating, for example, adhesion formation, pain, and fertility (https://drks.de/search/en/trial/DRKS00031857 (accessed on 1 October 2024)). Apart from those, studies in other gynecological fields such as hysterectomy and ovarian cancer would be desirable.

Another important aspect is that 4DryField PH can be an alternative to cauterization. Avoiding cauterization, for example, in the surgery for ovarian cysts, can prevent tissue trauma and support fertility. Two ongoing RCTs include patients with the unilateral or bilateral resection of ovarian cysts and compare cauterization and 4DryField PH for hemostasis. Outcomes include several fertility-related markers that are expected to provide insights into whether it represents the better treatment in such cases (https://drks.de/search/de/trial/DRKS00033772 (accessed on 1 October 2024)).

Future studies should focus further on patient reported outcomes, including quality of life and fertility rates, as well as the health economic benefits that could support broader reimbursement and adoption.

## Figures and Tables

**Table 1:** Tips and tricks for application of 4DryField PH.

Tips for Application as In Situ Gel: Hemostasis and Adhesion Prevention
Distribute evenly to a complete powder layer;
A white appearance on the surface shows complete hemostasis,
Generously drizzle with a sterile solution to transform all powder into a gel (e.g., saline, Ringer´s);
Avoid rinsing off the gel with high pressure, apply sterile solution gently;
Avoid soaking the powder with flushing fluid to create a fibrin-free gel;
Especially suitable for open surgery or laparoscopy with smaller and bleeding areas;
Helpful for larger areas with diffuse bleeding.
**Tips for Application as Premixed Gel: Adhesion Prevention**
A 10 mL sterile solution per 1 g of 4DryField PH fits many applications, but the ratio can be adapted individually;
Make sure to empty the bellows bottle completely by tapping on the bottom;
Mix until a homogeneous gel has formed;
Strictly avoid contamination with blood, e.g., from gloves;
Generously apply the gel to all desired areas;
Especially suitable for laparoscopic surgery with larger wounds, with little or no bleeding;
A more liquid gel may help cover larger areas.

## Data Availability

No new data were created or analyzed in this study. Data sharing is not applicable to this article.

## References

[1] Penzias A. (2019). Postoperative adhesions in gynecologic surgery: A committee opinion. Fertil. Steril..

[2] Koninckx P.R., Gomel V., Ussia A., Adamyan L. (2016). Role of the peritoneal cavity in the prevention of postoperative adhesions, pain, and fatigue. Fertil. Steril..

[3] Ahmad G., Kim K., Thompson M., Agarwal P., O’Flynn H., Hindocha A., Watson A. (2020). Barrier agents for adhesion prevention after gynaecological surgery. Cochrane Database Syst. Rev..

[4] Krielen P., Stommel M.W.J., Pargmae P., Bouvy N.D., Bakkum E.A., Ellis H., Parker M.C., Griffiths E.A., van Goor H., Broek R.P.G.T. (2020). Adhesion-related readmissions after open and laparoscopic surgery: A retrospective cohort study (SCAR update). Lancet.

[5] Krämer B., Neis F., Brucker S., Kommoss S., Andress J., Hoffmann S. (2021). Peritoneal Adhesions and their Prevention-Current Trends. Surg. Technol. Int..

[6] Menzies D., Ellis H. (1990). Intestinal obstruction from adhesions–how big is the problem?. Ann. R. Coll. Surg. Engl..

[7] Torres-de la Roche L.A., Catena U., Clark T., Devassy R., Leyland N., De Wilde R. (2023). Perspectives in adhesion prevention in gynaecological surgery. Facts Views Vis. Obgyn.

[8] Al-Sunaidi M., Tulandi T. (2006). Adhesion-related bowel obstruction after hysterectomy for benign conditions. Obstet. Gynecol..

[9] Arabkhazaeli M., Keltz J., Eisenberg R., Levie M., Luts H.Y. (2020). A Retrospective Study of Risk Factors for Small Bowel Obstruction After Hysterectomy. JSLS J. Soc. Laparosc. Robot. Surg..

[10] Mais V., Ajossa S., Marongiu D., Peiretti R.F., Guerriero S., Melis G.B. (1995). Reduction of adhesion reformation after laparoscopic endometriosis surgery: A randomized trial with an oxidized regenerated cellulose absorbable barrier. Obstet. Gynecol..

[11] Ten Broek R.P.G., Toneman M.K., van Goor H. (2023). [Adhesions after abdominal surgery: Developments in diagnosis and treatment]. Ned. Tijdschr. Geneesk.d.

[12] Tabibian N., Swehli E., Boyd A., Umbreen A., Tabibian J. (2017). Abdominal adhesions: A practical review of an often overlooked entity. Ann. Med. Surg..

[13] Ellis H., Moran B.J., Thompson J.N., Parker M.C., Wilson M.S., Menzies D., McGuire A., Lower A.M., Hawthorn R.J., O’Brien F. (1999). Adhesion-related hospital readmissions after abdominal and pelvic surgery: A retrospective cohort study. Lancet.

[14] Esber S., Etrusco A., Laganà A.S., Chiantera V., Arsalan H.M., Khazzaka A., Dellino M., Sleiman Z. (2023). Clinical Outcomes after the Use of Antiadhesive Agents in Laparoscopic Reproductive Surgery. Gynecol. Obstet. Investig..

[15] Ahmad G., Thompson M., Kim K., Agarwal P., Mackie F.L., Dias S., Metwally M., Watson A. (2020). Fluid and pharmacological agents for adhesion prevention after gynaecological surgery. Cochrane Database Syst. Rev..

[16] Hooker A.B., de Leeuw R.A., Emanuel M.H., Mijatovic V., Brolmann H.A.M., Huirne J.A. (2022). The link between intrauterine adhesions and impaired reproductive performance: A systematic review of the literature. BMC Pregnancy Childbirth.

[17] Dreisler E., Kjer J.J. (2019). Asherman’s syndrome: Current perspectives on diagnosis and management. Int. J. Women’s Health.

[18] Zhang W., French H., O’Brien M., Movilla P., Isaacson K., Morris S. (2023). Incidence of Intrauterine Adhesions After Hysteroscopic Myomectomy in Patients Seeking Fertility. J. Minim. Invasive Gynecol..

[19] De Wilde R.L., Brölmann H., Koninckx P.R., Lundorff P., Lower A.M., Wattiez A., Mara M., Wallwiener M. (2012). Prevention of adhesions in gynaecological surgery: The 2012 European field guideline. Gynecol. Surg..

[20] De Wilde R.L., Bakkum E.A., Brölmann H., Crowe A., Koninckx P., Korell M., Lundorff P., Pistofidis G., Tchartchian G., Trew G. (2014). Consensus recommendations on adhesions (version 2014) for the ESGE Adhesions Research Working Group (European Society for Gynecological Endoscopy): An expert opinion. Arch. Gynecol. Obstet..

[21] Torres-De La Roche L.A., Campo R., Devassy R., Sardo A.D.S., Hooker A., Koninckx P., Urman B., Wallwiener M., De Wilde R.L. (2019). Adhesions and Anti-Adhesion Systems Highlights. Facts Views Vis. Obgyn.

[22] Wiseman D.M., Trout J.R., Diamond M.P. (1998). The rates of adhesion development and the effects of crystalloid solutions on adhesion development in pelvic surgery. Fertil. Steril..

[23] Kramer B., Andress J., Neis F., Hoffmann S., Brucker S., Kommoss S., Höller A. (2021). Adhesion prevention after endometriosis surgery-results of a randomized, controlled clinical trial with second-look laparoscopy. Langenbecks Arch. Surg..

[24] Brochhausen C., Schmitt V.H., Planck C.N.E., Rajab T.K., Hollemann D., Tapprich C., Krämer B., Wallwiener C., Hierlemann H., Zehbe R. (2012). Current strategies and future perspectives for intraperitoneal adhesion prevention. J. Gastrointest. Surg..

[25] Ellis H., Harrison W., Hugh T.B. (1965). The healing of peritoneum under normal and pathological conditions. Br. J. Surg..

[26] Shapiro L., Holste J.-L., Muench T., Dizerega G. (2015). Rapid reperitonealization and wound healing in a preclinical model of abdominal trauma repair with a composite mesh. Int. J. Surg..

[27] Schaefer S.D., Alkatout I., Dornhoefer N., Herrmann J., Klapdor R., Meinhold-Heerlein I., Meszaros J., Mustea A., Oppelt P., Wallwiener M. (2024). Prevention of peritoneal adhesions after gynecological surgery: A systematic review. Arch. Gynecol. Obstet..

[28] Sieg L., Eismann H., Schumacher C., Flöricke F., Johanning K., Albrecht A.A. (2017). Effects of Microporous Polysaccharide Powder in a Model of Dilution on Viscoelastic Characteristics of Clot Formation—An In-Vitro Study. ARC J. Anesthesiol..

[29] Hanke A.A., Flöricke F., Sieg L., Johanning K., Rahe-Meyer N. (2011). Effects of a New Microporous Polysaccharide Powder on Viscoelastic Characteristics of Clot Formation. Anesthesiology 2011, Proceedings of the American Society of Anesthesiologists Annual Meeting, Chicago, IL, USA, 15–19 October 2011.

[30] Poehnert D., Abbas M., Maegel L., Sambale F., Lavrentieva A., Kreipe H.-H., Klempnauer J., Winny M. (2015). Evaluation of the biological tolerability of the starch-based medical device 4DryField^®^ PH in vitro and in vivo a rat model. J. Biomater. Appl..

[31] Cesnjevar R., Purbojo A., Haake C., Laas J. (2022). Significant adhesion reduction and time saving in pediatric heart surgery with 4DryField PH: A retrospective, controlled study. PLoS ONE.

[32] Ziegler N., la Roche L.A.T.-D., Devassy R., De Wilde R.L. (2021). Changed inflammatory markers after application of 4DryField PH for adhesion prevention in gynecological surgery. Arch. Gynecol. Obstet..

[33] Korell M., Ziegler N., De Wilde R.L. (2016). Use of Modified Polysaccharide 4DryField^®^ PH for Adhesion Prevention and Hemostasis in Gynecological Surgery: A Two-Center Observational Study by Second-Look Laparoscopy. Biomed. Res. Int..

[34] Poehnert D., Abbas M., Kreipe H.-H., Klempnauer J., Winny M. (2015). Evaluation of 4DryField^®^ PH as Adhesion Prevention Barrier Tested in an Optimized Adhesion Model (OPAM) in Rats. Eur. Surg. Res..

[35] Ziegler N., Korell M., Herrmann A., de Wilde M.S., la Roche L.A.T.-D., Larbig A., De Wilde R.L. (2016). Uterine perforation following a fractional curettage successfully treated with the modified polysaccharide 4DryField^®^ PH: A case report. J. Med. Case Rep..

[36] Lisa Z., Richtarova A., Hlinecka K., Boudova B., Kuzel D., Fanta M., Mara M. (2024). 4DryField vs. hyalobarrier gel for preventing the recurrence of intrauterine adhesions—a pilot study. Minim. Invasive Ther. Allied Technol..

[37] Kraemer B., Andress J., Neis F., Hoffmann S., Brucker S., Kommoss S., Höller A. (2023). Improvement in Fertility and Pain after Endometriosis Resection and Adhesion Prevention with 4DryField(^®^) PH: Follow-up of a Randomized Controlled Clinical Trial. J. Clin. Med..

[38] Blumhardt G., Haas M., Polte S. (2018). Effect of 4DryField^®^ PH, a Novel Adhesion Barrier, on Recurrence of Intestinal Adhesions after Extensive Visceral Adhesiolysis. Case Rep. Surg..

[39] Ziegler N., De Wilde R.L. (2021). Reduction of adhesion formation after gynaecological adhesiolysis surgery with 4DryField PH—a retrospective, controlled study with second look laparoscopies. J. Obstet. Gynaecol..

[40] lkatout I., Wedel T., Pape J., Possover M., Dhanawat J. (2021). Review: Pelvic nerves-from anatomy and physiology to clinical applications. Transl. Neurosci..

[41] Allahqoli L., Hakimi S., Momenimovahed Z., Mazidimoradi A., Rezaei F., Aghamohammadi S.Z., Rahmani A., Mansouri G., Hadavandsiri F., Salehiniya H. (2024). Neuropelveology for Endometriosis Management: A Systematic Review and Multilevel Meta-Analysis. J. Clin. Med..

[42] Deckers P., Ribeiro S.C., Simões R.d.S., Miyahara C.B.d.F., Baracat E.C. (2018). Systematic review and meta-analysis of the effect of bipolar electrocoagulation during laparoscopic ovarian endometrioma stripping on ovarian reserve. Int. J. Gynaecol. Obstet..

[43] Younis J.S., Taylor H.S. (2024). The impact of ovarian endometrioma and endometriotic cystectomy on anti-Müllerian hormone, and antral follicle count: A contemporary critical appraisal of systematic reviews. Front. Endocrinol..

[44] Watrowski R. (2020). Unifying local hemostasis and adhesion prevention during gynaecologic laparoscopies: Experiences with a novel, plant-based agent. J. Obstet. Gynaecol..

[45] Torres-de la Roche L.A., Devassy R., de Wilde M.S., Cezar C., Krentel H., Korell M., De Wilde R.L. (2020). A new approach to avoid ovarian failure as well function-impairing adhesion formation in endometrioma infertility surgery. Arch. Gynecol. Obstet..

[46] Moszynski R., Burchardt B., Sajdak S., Moszynska M., Englert-Golon M., Olbromski P. (2022). Using a Modified Polysaccharide as a Hemostatic Agent Results in Less Reduction of the Ovarian Reserve after Laparoscopic Surgery of Ovarian Tumors-Prospective Study. Medicina.

[47] Toneman M., Groenveld T., Krielen P., Hooker A., de Wilde R., la Roche L.A.T.-D., Sardo A.D.S., Koninckx P., Cheong Y., Nap A. (2023). Risk Factors for Adhesion-Related Readmission and Abdominal Reoperation after Gynecological Surgery: A Nationwide Cohort Study. J. Clin. Med..

[48] Güven E., Dura M.C., Aktürk H., Güraslan H. (2023). Safety of Laparoscopic Entry Points in Patients With a History of Abdominal Surgery: A Research Article. Cureus.

[49] Gluck O., Mizrachi Y., Kovo M., Divon M., Bar J., Weiner E. (2017). Major underestimation and overestimation of visual blood loss during cesarean deliveries: Can they be predicted?. Arch. Gynecol. Obstet..

[50] Shenhav S., Grin L., Kapustian V., Anteby E.Y., Gdalevich M., Gemer O. (2019). Quantifying the effects of postcesarean adhesions on incision to delivery time. J. Matern. Fetal Neonatal Med..

[51] Wright J.D., Huang Y., Li A.H., Melamed A., Hershman D.L. (2022). Nationwide Estimates of Annual Inpatient and Outpatient Hysterectomies Performed in the United States. Obstet. Gynecol..

[52] Pickett C.M., Seeratan D.D., Mol B.W.J., Nieboer T.E., Johnson N., Bonestroo T., Aarts J.W.M. (2023). Surgical approach to hysterectomy for benign gynaecological disease. Cochrane Database Syst. Rev..

[53] Brandsborg B. (2012). Pain following hysterectomy: Epidemiological and clinical aspects. Dan. Med. J..

[54] Betcher R.E., Chaney J.P., Lacy P.R., Otey S.K., Wood D.J. (2013). Analysis of postoperative pain in robotic versus traditional laparoscopic hysterectomy. J. Robot. Surg..

[55] Khapre S.S., Joshi V., Hivre M.D. (2024). Hysterectomy Profile in King Edward Memorial Hospital, Pune, India: Indications, Routes of Surgery, and Complications. Cureus.

[56] Yang J.-H., Chen M.-J., Chen C.-D., Chen S.-U., Ho H.-N., Yang Y.-S. (2013). Optimal waiting period for subsequent fertility treatment after various hysteroscopic surgeries. Fertil. Steril..

[57] Valle R.F., Sciarra J.J. (1988). Intrauterine adhesions: Hysteroscopic diagnosis, classification, treatment, and reproductive outcome. Am. J. Obstet. Gynecol..

[58] Sardo A.D.S., Spinelli M., Bramante S., Scognamiglio M., Greco E., Guida M., Cela V., Nappi C. (2011). Efficacy of a polyethylene oxide-sodium carboxymethylcellulose gel in prevention of intrauterine adhesions after hysteroscopic surgery. J. Minim. Invasive Gynecol..

[59] Karsch J.-J., Berthold M., Breul J. (2016). Evaluation of Lymphorrhea and Incidence of Lymphoceles: 4DryField^®^ PH in Radical Retropubic Prostatectomy. Adv. Urol..

[60] Poehnert D., Neubert L., Winny M. (2024). Comparison of adhesion prevention capabilities of the modified starch powder-based medical devices 4DryField((R)) PH, HaemoCer PLUS and StarSil((R)) in the Optimized Peritoneal Adhesion Model. Int. J. Med. Sci..

[61] Poehnert D., Neubert L., Winny M. (2019). Comparison of adhesion prevention capabilities of the modified starch powder-based medical devices 4DryField((R)) PH and Arista AH in the Optimized Peritoneal Adhesion Model. Int. J. Med. Sci..

[62] Akasaka H., Naora H. (2023). Revisiting the Use of Normal Saline for Peritoneal Washing in Ovarian Cancer. Int. J. Mol. Sci..

[63] Akasaka H., Lee W., Ko S.Y., Lengyel E., Naora H. (2023). Normal saline remodels the omentum and stimulates its receptivity for transcoelomic metastasis. JCI Insight.

[64] van Westreenen M., Tol P.v.D., Pronk A., Marquet R., Jeekel J., Leguit P. (1999). Perioperative lavage promotes intraperitoneal adhesion in the rat. Eur. Surg. Res..

[65] Cwalinski J., Staniszewski R., Baum E., Jasinski T., Mackowiak B., Bręborowicz A. (2015). Normal saline may promote formation of peritoneal adhesions. Int. J. Clin. Exp. Med..

[66] Ahmad M., Crescenti F. (2019). Significant Adhesion Reduction with 4DryField PH after Release of Adhesive Small Bowel Obstruction. Surg. J..

[67] Cox K.R. (1970). Starch granuloma (pseudo-malignant seedlings). Br. J. Surg..

[68] Ellis H. (1994). Pathological changes produced by surgical dusting powders. Ann. R. Coll. Surg. Engl..

[69] Ereth M.H., Schaff M., Ericson E.F., Wetjen N.M., Nuttall G.A., Oliver W.C. (2008). Comparative Safety and Efficacy of Topical Hemostatic Agents in a Rat Neurosurgical Model. Neurosurgery.

[70] Velasquez D., Pavon-Djavid G., Chaunier L., Meddahi-Pellé A., Lourdin D. (2015). Effect of crystallinity and plasticizer on mechanical properties and tissue integration of starch-based materials from two botanical origins. Carbohydr. Polym..

[71] Liu T.-M., Kiu K.-T., Yen M.-H., Tam K.-W., Chang T.-C. (2023). Efficacy and safety of purified starch for adhesion prevention in colorectal surgery. Heliyon.

[72] Vrijland W.W., Tseng L.N.L., Eijkman H.J.M., Hop W.C.J., Jakimowicz J.J., Leguit P., Stassen L.P.S., Swank D.J., Haverlag R., Bonjer H.J. (2002). Fewer intraperitoneal adhesions with use of hyaluronic acid-carboxymethylcellulose membrane: A randomized clinical trial. Ann. Surg..

[73] Hajibandeh S., Saeed S., Bird J., Kannappa L., Ratnayake I. (2022). Effect of hyaluronate-based bioresorbable membrane (Seprafilm) on outcomes of abdominal surgery: A meta-analysis and trial sequential analysis of randomised controlled trials. Updates Surg..

[74] Beck D.E., Cohen Z., Fleshman J.W., Kaufman H.S., van Goor H., Wolff B.G. (2003). A prospective, randomized, multicenter, controlled study of the safety of Seprafilm adhesion barrier in abdominopelvic surgery of the intestine. Dis. Colon. Rectum.

[75] Lee W.S., Baek J.H., Lee W.K. (2013). Direct comparison of Seprafilm(R) versus Adept (R) versus no additive for reducing the risk of small-bowel obstruction in colorectal cancer surgery. Surg. Today.

[76] Stoerzer S., Winny M., Beetz O., Jacobi S., Klempnauer J., Poehnert D. (2024). Impact of the starch-based anti-adhesive agent 4DryField PH on anastomotic healing after rectal surgery. Int. J. Surg. Open.

